# Interleukin-13 in Asthma and Other Eosinophilic Disorders

**DOI:** 10.3389/fmed.2017.00139

**Published:** 2017-09-19

**Authors:** Emma Doran, Fang Cai, Cécile T. J. Holweg, Kit Wong, Jochen Brumm, Joseph R. Arron

**Affiliations:** ^1^Immunology Discovery, Genentech, Inc., South San Francisco, CA, United States; ^2^OMNI Biomarker Development, Genentech, Inc., South San Francisco, CA, United States; ^3^Biostatistics, Genentech, Inc., South San Francisco, CA, United States

**Keywords:** interleukin-13, eosinophils, chemokines, asthma, eosinophilic disorders

## Abstract

Asthma is characterized by episodic, reversible airflow obstruction associated with variable levels of inflammation. Over the past several decades, there has been an increasing appreciation that the clinical presentation of asthma comprises a diverse set of underlying pathologies. Rather than being viewed as a single disease entity, asthma is now thought of as a clinical syndrome with the involvement of multiple pathological mechanisms. While it is appreciated that eosinophilia is present in only a subset of patients, it remains a key feature of asthma and other eosinophilic disorders such as atopic dermatitis, eosinophilic esophagitis, and chronic rhinosinusitis with nasal polyps. Eosinophils are bone marrow-derived leukocytes present in low numbers in health; however, during disease the type 2 cytokines [interleukins (IL)-4, -5, and -13] can induce rapid eosinophilopoiesis, prolonged eosinophil survival, and trafficking to the site of injury. In diseases such as allergic asthma there is an aberrant inflammatory response leading to eosinophilia, tissue damage, and airway pathology. IL-13 is a pleiotropic type 2 cytokine that has been shown to be integral in the pathogenesis of asthma and other eosinophilic disorders. IL-13 levels are elevated in animal models of eosinophilic inflammation and in the blood and tissue of patients diagnosed with eosinophilic disorders. IL-13 signaling elicits many pathogenic mechanisms including the promotion of eosinophil survival, activation, and trafficking. Data from preclinical models and clinical trials of IL-13 inhibitors in patients have revealed mechanistic insights into the role of this cytokine in driving eosinophilia. Promising results from clinical trials further support a key mechanistic role of IL-13 in asthma and other eosinophilic disorders. Here, we provide a perspective on the role of IL-13 in asthma and other eosinophilic disorders and describe ongoing clinical trials targeting this pathway in patients with significant unmet medical needs.

## Introduction

Eosinophils are bone marrow-derived leukocytes that are present in low numbers in the blood during health (typically < 5% of all white blood cells) and rapidly migrate to select tissues where they reside. However, increased blood and tissue eosinophil counts have been associated with multiple pathologies. During parasitic infection and allergic diseases rampant eosinophilopoiesis occurs leading to increased numbers in the peripheral blood. Eosinophils then become activated and migrate to the site of injury where they can release mediators, including cytokines, chemokines, and cytotoxic granule proteins. This ultimately leads to parasite expulsion, or in the case of allergic diseases, tissue injury (1).

Investigation of mouse models and human disease has found that increased eosinophil numbers are associated with type 2 inflammation and an increase of interleukin (IL)-4, -5, and -13. Indeed, eosinophilic disorders are predominantly characterized by type 2 inflammation. IL-13 is a pleiotropic type 2 cytokine that has been shown to be important in the pathogenesis of asthma and other eosinophilic disorders. The effects of IL-13 in these conditions include induction of goblet cell metaplasia and increased mucus secretion, increased airway hyperreactivity, and, indirectly, trafficking of eosinophils to the site of tissue injury *via* chemotaxis (2).

The prevalence of eosinophilic syndromes is continuing to increase with more severe forms of disease refractory to standard of care thus necessitating a better understanding of underlying biology to enable the development of new treatments. Therapeutics targeting type 2 inflammation, including IL-4, IL-5, and IL-13, are currently in development to treat eosinophilic diseases. However, due to the overlapping biology of these cytokines it has been a challenge to delineate the exact roles each play in type 2/eosinophilic disease. Here, we provide a review of the literature describing the role of IL-13 and the ongoing clinical development of therapeutics targeting IL-13 in asthma and other eosinophilic disorders such as atopic dermatitis (AD), eosinophilic esophagitis (EoE), and chronic rhinosinusitis (CRS) with nasal polyps (CRSwNP).

## Inflammation in Eosinophilic Diseases

Eosinophils develop from pluripotent progenitors in bone marrow and migrate into peripheral blood once mature. Mature eosinophils have distinct bilobed nuclei and secretory granules allowing them to be easily identified by routine tissue histology using hematoxylin and eosin staining. Eosinophils are terminal cytotoxic effector cells and make unique contributions to both innate and adaptive immunity (3). They have a half-life of ~18 h in blood and under homeostatic conditions quickly migrate to spleen, lymph nodes, thymus, gastrointestinal tract, uterus, and mammary glands, recruited by chemotactic factors (4). The evolutionary function of type 2 inflammation is primarily to respond to and control infection by extracellular parasitic organisms. Infection with parasitic worms elicits a Th2-mediated response that is required for the successful expulsion of the parasitic burden and protection of the host. Classical Th2 effector mechanisms are employed to expel the infectious organisms including mastocytosis, eosinophilia, increased mucus production, smooth muscle hypercontractility, and IgE synthesis. At the site of infection, eosinophils degranulate releasing cytotoxic granules to assist with killing of the parasite. They also secrete many mediators including IL-4 and IL-13 to perpetuate further type 2 inflammation (5, 6).

However, the presence of eosinophils in classic type 2 diseases such as asthma, AD, EoE, and CRSwNP can be pathogenic. The relationship between the presence of eosinophils in tissue and pathology has long been established, as seen in postmortem examinations of patients who suffered from fatal asthma exacerbations (7). In the instance of asthma, there is an aberrant response to non-parasite triggers such as allergens, viruses, or mucosal injury leading to epithelial cells producing cytokines, including IL-25, IL-33, thymic stromal lymphopoietin (TSLP), and IL-1α. These so-called type 2 alarmins can then promote differentiation of T helper 2 (Th2) cells, as well as activation of mast cells, macrophages, and type 2 innate lymphoid cells (ILC2s). IL-4, IL-5, and IL-13 secreted from these cells can subsequently elicit further immune activation including eosinophilic responses. IL-5 is the major cytokine responsible for eosinophilopoiesis, along with granulocyte-macrophage colony-stimulating factor (GM-CSF) and IL-3, which also support eosinophil survival (8, 9). The role of IL-5 in eosinophilic diseases is reviewed elsewhere in this issue.

Human IL-13 was first discovered in 1993 and has since been shown to be produced by multiple cell types. Increased IL-13 expression can elicit many of the pathological findings associated with type 2 diseases (10). The functions of IL-13 *in vivo* were elucidated by the generation of a mouse strain selectively overexpressing IL-13 in the lung *via* a transgene regulated by the club cell-specific CC10 promoter (11). This airway-specific IL-13 transgenic mouse presented with eosinophilic lung inflammation, airway epithelial cell hypertrophy, goblet cell metaplasia, mucus hypersecretion, subepithelial fibrosis, and airway hyperresponsiveness (AHR). In an ovalbumin (OVA) challenge model, IL-13 was found to be essential for the maintenance of AHR and mucus hypersecretion as administration of an IL-13 neutralizing antibody resulted in attenuation of these responses (12). ILC2s were found to expand *in vivo* in response to the innate type 2 cytokines IL-25 and IL-33 and represented the predominant early source of IL-13 during *Nippostrongylus brasiliensis* infection to allow for efficient helminth expulsion (13).

**Figure 1:**
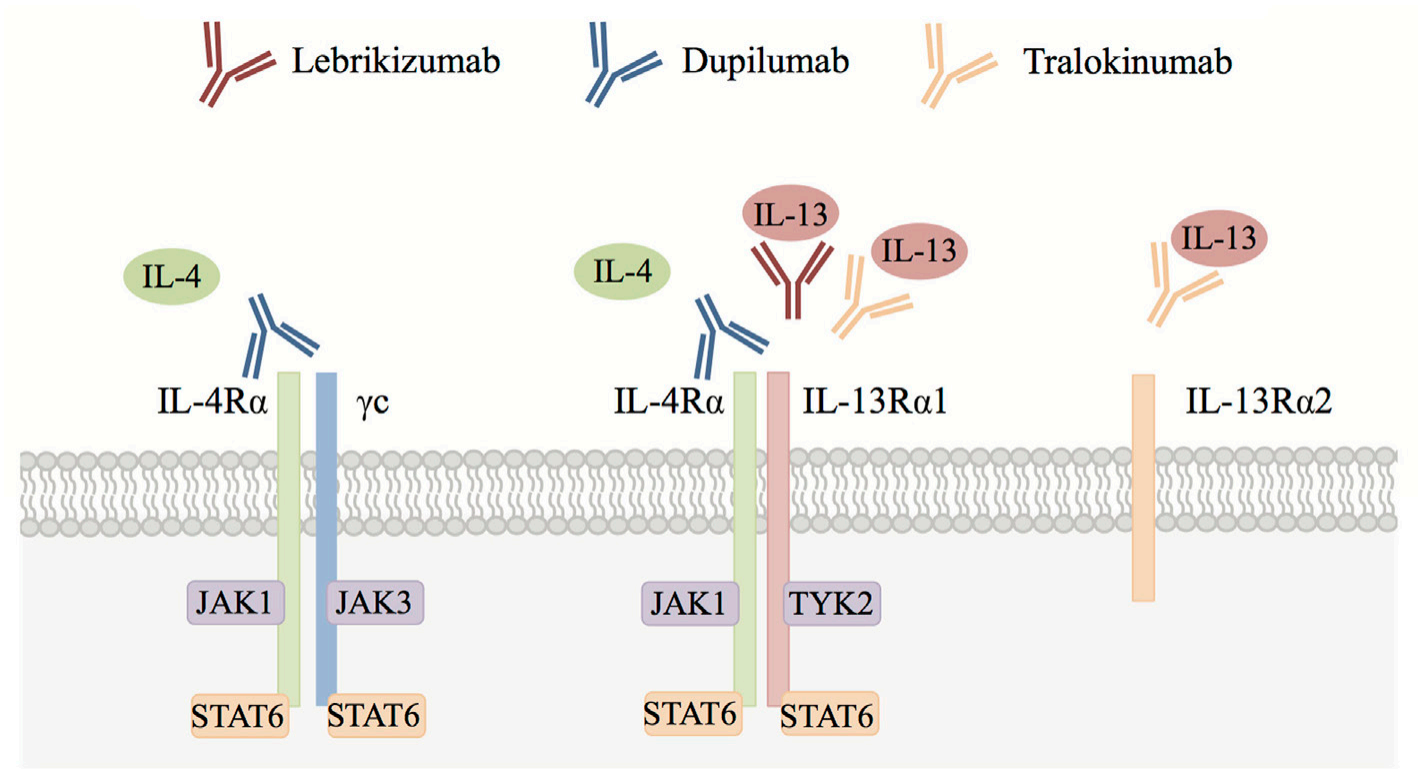
Interleukin (IL)-4/IL-13 cytokine signaling. IL-4 signals through both the IL-4 receptor α (IL-4Rα)/γc (type I) and IL-4Rα/IL-13Rα1 (type II) receptor complexes, whereas IL-13 signals only through the IL-4Rα/IL-13Rα1 receptor complex. IL-13 can also bind to the IL-13Rα2 chain, which is thought to act primarily as a decoy receptor. Both IL-4 and IL-13 activate signal transducer and activator of transcription 6 (STAT6) via Janus kinase (JAK) family kinases leading to type 2 responses and eosinophilic inflammation in tissues orchestrated by chemokines, growth factors, and factors that position eosinophils in the tissue (see text for details). Blocking antibodies including lebrikizumab, dupilumab, and tralokinumab have been developed to inhibit IL-4 and/or IL-13 signaling in eosinophilic diseases.

Interestingly, IL-4 and IL-13 both signal through the IL-4 receptor α (IL-4Rα). IL-4Rα is a component of the type I (IL-4Rα and γc) and type II (IL-4Rα and IL-13Rα1) IL-4R complexes. IL-4 can signal through both type I and II receptor complexes, whereas IL-13 signals only through the type II receptor complex. IL-4 and IL-13 activate the Janus kinase–signal transducer and activator of transcription (JAK-STAT) pathway. For example, IL-13 engages with its cell surface receptor IL-13Rα1 that then associates with IL-4Rα resulting in phosphorylation of JAK1 and TYK2. These activated kinases then phosphorylate the cytoplasmic domain of the receptor, creating binding sites for STAT6. STAT6 molecules are in turn phosphorylated, whereupon they dimerize and translocate to the nucleus. There they regulate gene transcription, ultimately leading to the production of type 2 cytokines such as IL-13, eotaxins, and other mediators involved in eosinophilic inflammation (Figure 1) (14). IL-4Rα and IL-13Rα1 are expressed on both hematopoietic and non-hematopoietic cells such as macrophages, B cells, fibroblasts, and airway epithelial cells. It is thought that this receptor configuration is responsible for the fact that IL-4 and IL-13 have overlapping functions as well as the ability to act independently of each other. For example, IL-4 alone has been implicated in initiating and potentiating polarization of naive T cells to Th2 cells and has a more dominant role than IL-13 in antibody class switching to IgE (15). IL-13 on the other hand plays a key role in fibrosis and mucus secretion (2). These distinct functions may be due to both differential expression of type I and type II receptor complexes and differential spatiotemporal secretion of IL-4 and IL-13. However, both IL-4 and IL-13 can contribute to inflammation, AHR, and induction of chemokines that drive chemotaxis of blood eosinophils to injured tissue (16). Of note, IL-13 can also bind to the IL-13Rα2 chain, which does not contain a transmembrane-signaling domain and thus is thought to primarily act as a decoy receptor (17, 18).

**Figure 2:**
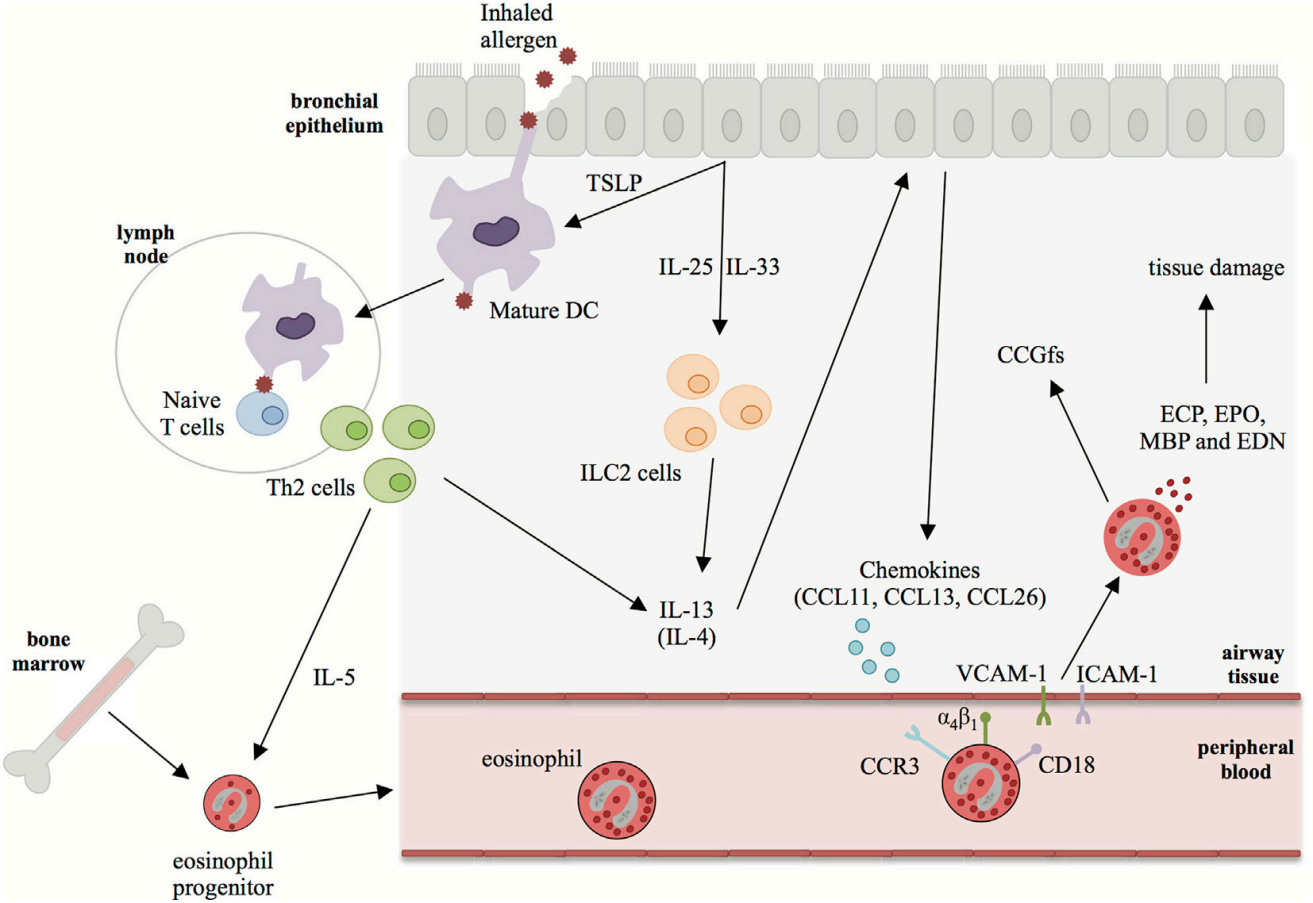
The role of interleukin (IL)-13 in driving eosinophilia in asthma. In asthma, bronchial epithelial cell injury leads to production of type 2 alarmins such as IL-25, IL-33, and thymic stromal lymphopoietin (TSLP). These alarmins can promote the differentiation of Th2 cells and activation of ILC2s leading to production of IL-4, IL-5, and IL-13. IL-5 is the major cytokine responsible for eosinophilopoiesis and eosinophil survival in the periphery. IL-13 (and, to a lesser extent, IL-4) induces the secretion of chemokines, such as CCL11, CCL13, and CCL26 from epithelial cells. Chemokines bind to CCR3 on eosinophils trafficking them to the site of injury where they extravasate into the lung tissue. In the lung eosinophils degranulate causing tissue damage *via* the secretion of eosinophil granule proteins and cytokines, chemokines, and growth factors (CCGfs).

In eosinophilic disorders such as asthma, there is increased eosinophilopoiesis and subsequent migration of eosinophils to the lung due to: (i) elevated levels of IL-3, IL-5, and GM-CSF to stimulate eosinophil development in bone marrow and survival in the blood and (ii) increased levels of type 2 cytokines (IL-4 and IL-13) to upregulate chemokine production, including CCL11 (eotaxin 1), CCL24 (eotaxin 2), CCL26 (eotaxin 3), CCL13 (MCP4), and CCL5 (RANTES), which enhance chemotaxis for eosinophil trafficking from the circulation to the airway (19). These chemokines bind to the chemokine receptor, CCR3, activating adhesion molecules such as integrins on the surface of blood eosinophils. In turn, this allows eosinophils to interact with endothelial cells *via* intracellular adhesion molecule-1 (ICAM-1), vascular cell adhesion molecule-1 (VCAM-1), and periostin leading to infiltration from blood to the airway tissue (20) (Figure 2). Chemokine knockout mice such as *CCL11*^−/−^ and *CCL24*^−/−^ show decreased trafficking of eosinophils to the airway during allergen challenge (21, 22). In an *Aspergillus fumigatus*-induced asthma model, CCR3 knockout mice had decreased eosinophilic airway inflammation along with reduced levels of type 2 cytokines, including IL-13 (23).

Two eosinophil-deficient mouse strains have been developed (ΔdblGATA and PHIL), however, studies inducing allergic airway inflammation in these models have reported conflicting results. OVA-challenged ΔdblGATA (24) mice had similar airway hyperreactivity and airway inflammation but reduced collagen deposition and airway smooth muscle mass compared to WT mice (25). However, OVA-challenged PHIL mice were protected from airway hyperreactivity and goblet cell metaplasia and mucus secretion (26). In another study, ΔdblGATA mice were protected from *A. fumigatus*-induced allergic airway inflammation and had decreased type 2 cytokines and airway mucus production (23). However, there are numerous caveats comparing these studies including the different strains of mice, variations in the models used, and disparities in experimental readouts. Further investigation is required to definitively characterize the relationship between IL-13 and eosinophils in mouse models of allergic disease.

Multiple biologics and small molecule therapeutic candidates targeting eosinophilic inflammation have been or are currently being evaluated in preclinical or clinical settings. A number of biologics blocking soluble inflammatory mediators and their receptors associated with eosinophilic inflammation, including IgE, IL-4, IL-4Rα, IL-5, IL-5R, IL-13, TSLP, IL-25, and IL-33, are being investigated. Anti-Siglec-8 antibodies have been proposed to inhibit eosinophil activation and induce eosinophil apoptosis (27). The advantages and potential limitations of different targeted therapies for eosinophilic disorders have been recently reviewed elsewhere (28).

## Asthma

Asthma is one of the most common chronic disorders in the world. Despite a vast body of research and the large clinical burden associated with asthma, its complexity and heterogeneity make it difficult to establish a standardized definition of what constitutes “disease.” Asthma is typically characterized by airway inflammation and a history of respiratory symptoms (including wheeze, shortness of breath, chest tightness, and cough) together with a variable airflow limitation (29).

Investigation into the complexity of asthma has led to the identification of multiple different clinical and molecular phenotypes (30). The most commonly described clinical phenotypes include those defined by severity, rates of exacerbations, response to treatment, age of onset, and obesity. The current molecular phenotypes include type 2/eosinophilic, neutrophilic, and paucigranulocytic inflammation (30, 31). These clinical and molecular phenotypes are not mutually exclusive, may change over time in individual patients, and may interact, contributing to differences in responsiveness to asthma therapies.

### Role of Eosinophils in Asthma Pathophysiology

Arguably, molecular phenotyping of asthma patients has been most valuable in developing novel targeted therapies, particularly in understanding the biology of type 2/eosinophilic asthma. Eosinophils have been observed in increased numbers in peripheral blood, bronchoalveolar lavage (BAL) fluid, and bronchial tissue in asthma patients. It has also been reported that elevated eosinophil counts are significantly correlated with disease severity, indicating that these cells may play an important role in asthma pathogenesis (32). Measurement of eosinophils in induced sputum has been shown to be a biomarker of airway inflammation and a useful tool for adjusting the intensity of corticosteroid treatment to achieve optimal asthma control (33–35). However, measurement of sputum eosinophils is not widely used in the clinical setting, as it is time-consuming, requires specialized technical expertise, and the collection process may cause some discomfort to patients. Elevated blood eosinophil counts are correlated with lung function and asthma symptom scores, and therefore, can be useful in both the diagnosis and the management of patients with asthma (32, 36–43). A statistically significant correlation between blood eosinophils and sputum eosinophils in asthma patients has been reported (44). Another study later showed that blood eosinophil counts could accurately predict airway eosinophilia in asthma patients with persistent uncontrolled disease despite treatment (45). Therefore, blood eosinophils may be a good surrogate biomarker to identify patients with airway eosinophilia. However, Wenzel et al. described two subtypes of asthma, eosinophilic and non-eosinophilic, with different pathological, physiological, and clinical characteristics, although it should be noted that these characteristics exist along a continuum rather than being completely independent. In their study, the presence of eosinophils in bronchial biopsies was associated with significantly increased tissue lymphocytes, mast cells, and macrophages, basement membrane thickening, and patient intubations compared to the non-eosinophilic asthmatics (46). Due to disease heterogeneity in poorly controlled asthma patients, there was a need to develop biomarkers that could enable the identification of a particular subset of patients. This has been valuable in recent clinical trials, for example, blood eosinophil counts correlated with response to anti-IL-13 therapies in Phase 2 clinical trials, wherein patients with higher levels of blood eosinophils had a greater benefit than patients with lower counts (47–50). In addition to blood eosinophil counts there are other biomarkers of type 2 diseases, such as serum periostin and fractional exhaled nitric oxide (FeNO), which are being investigated in asthma. Measurement of these biomarkers in a population of asthmatics revealed that they are continuously distributed and correlated with each other (44). This continuous distribution of biomarkers and airway pathology is a critical nuance to appreciate when interpreting clinical data, as cutoffs defining “biomarker-high” vs. “biomarker-low” populations are arbitrary and typically fall near the median of continuously distributed values rather than defining clear distinctions between subgroups (51).

### IL-13 and Eosinophils in Asthma

Interleukin-13 has been implicated in promoting eosinophil survival, activation, and recruitment. *In vitro* cultures of eosinophils with recombinant IL-13 showed prolonged survival in a dose-dependent manner, which was attributed to inhibition of apoptosis. This was mediated by an autocrine mechanism through stimulation or release of IL-3 and GM-CSF by eosinophils. A major function of IL-13 (and IL-4) in the asthmatic airway is to induce chemotaxis of eosinophils to the site of injury. A number of *in vitro* studies have investigated the role of IL-13-induced chemotaxis and activation of eosinophils. Significant dose-dependent chemotactic activity was observed in an experiment in which eosinophils were cultured in the upper compartment of chemotactic chambers with recombinant IL-13 in the lower compartment (52). *In vitro* culture of eosinophils stimulated with IL-13 resulted in a concentration-dependent upregulation of the activation marker CD69. Furthermore, the addition of an anti-IL-13 antibody to these cultures led to inhibition of this activation (53). IL-13 induces VCAM-1 expression in endothelial cells, leading to increased adhesiveness of eosinophils to endothelium via VCAM-1/integrin α4 interactions. This might be a potential mechanism by which IL-13 promotes arrest and extravasation of eosinophils to the asthmatic airway (54). In clinical studies, increased IL-13 mRNA expression in sputum specimens and bronchial mucosa was significantly positively correlated with the percentage of eosinophils in the airway lumen (55, 56). In a study of human bronchial epithelial cells from asthma patients, an IL-13-inducible gene signature (*POSTN, CLCA1*, and *SERPINB2*) was identified that served as a surrogate marker of type 2 airway inflammation. This signature was observed in about half of asthmatics and was associated with distinct features of asthma including airway eosinophilia (56). In addition, eosinophils in the bronchial submucosa were found to express IL-13 (57). IL-13 is produced and consumed locally at sites of inflammation, therefore, peripheral levels are very low, and developing reliable assays to measure circulating IL-13 has been an on-going challenge. Recently, we developed an assay to detect human serum IL-13 with femtograms per milliliter sensitivity and excellent specificity (58). Using this assay, we found significantly higher levels of serum IL-13 in severe asthma patients relative to healthy volunteers and these levels strongly correlated with the type 2 gene signature in bronchial epithelium. Interestingly, in moderate to severe asthma patients, serum IL-13 was strongly positively correlated with blood eosinophil counts. It has also been demonstrated that human eosinophils derived from both periphery and tissue are capable of synthesizing, and upon stimulation releasing over 35 cytokines, chemokines, and growth factors. IL-13 is an abundant cytokine in eosinophils and upon release may directly orchestrate inflammatory responses with other immunomodulators (59). It is therefore possible that the elevated serum IL-13 levels may be in part a consequence of release from both airway and blood eosinophils (58).

### IL-13 and Eosinophil Activation in Asthma

Type 2 cytokines, including IL-13, regulate the secretion of various chemokines that can bind to eosinophils *via* the CCR3 receptor leading to eosinophil activation and migration to the lung *via* chemotaxis. Eosinophils recruited to the asthmatic airway are highly activated and localize with inflammatory mediators and other immune cells that accumulate at the site of injury. While eosinophils can secrete cytokines and other mediators without degranulating, the ultimate result of eosinophil activation is degranulation. Human eosinophil granules contain four cationic proteins, major basic protein (MBP) primarily present in the crystalline core, eosinophil peroxidase (EPO/EPX), eosinophil cationic protein (ECP), and eosinophil-derived neurotoxin (EDN) enriched in the granule matrix. The secretion of eosinophil granule proteins has been shown to facilitate the killing of parasites; *in vitro* ECP and MBP were found to be toxic to the larvae of parasites such as *Schistosoma mansoni* and *Trichinella spiralis* (60). However, they also have cytotoxic effects on tissues and their levels have been suggested to be associated with asthma severity, bronchial epithelial cell damage, and remodeling (61).

The most commonly observed forms of eosinophil degranulation in the inflammatory airway are piecemeal degranulation and cytolytic degranulation. Piecemeal degranulation is a form of exocytosis, in which specific granule contents are transported to the cell surface in small cytoplasmic secretory vesicles, while cells remain viable (62). Cytolytic degranulation on the other hand involves eosinophil chromatolysis and cell membrane rupture leading to the release of intact secretory granules. Cell-free eosinophil granules can store and further release their contents (61). One study has described eosinophil granules in airway macrophages that had presumably phagocytosed apoptotic eosinophils. Increasing numbers of macrophages containing ECP and EPO were observed with increasing severity of asthma (63). Besides the most common piecemeal and cytolytic degranulation, another form of eosinophil degranulation is to generate extracellular traps (ETosis) containing granule proteins in response to exposure to bacteria, C5a, or CCL11 (64, 65). The process of eosinophil degranulation in the peripheral blood is less clearly defined, and there have been conflicting reports as to whether peripheral blood eosinophil degranulation contributes to disease. It has been shown that blood eosinophils in allergic diseases such as asthma and AD display no morphological signs of either piecemeal or cytolytic degranulation while eosinophils from matched diseased tissue biopsies exhibited degranulation through both piecemeal and cytolytic processes. These observations suggest that eosinophils exist in a resting status in the circulation and are activated at the site of tissue pathology (66). However, a small study with mild allergic asthmatics and healthy volunteers demonstrated that blood eosinophils in allergic patients underwent piecemeal degranulation during pollen season (67). A study in children comparing blood eosinophils from healthy controls, symptom-free asthmatics, and asthmatics with acute exacerbations showed that the proportion of activated blood eosinophils with significant morphologic changes were highest in the children with acute asthma exacerbations compared to symptom-free asthmatics and healthy controls (68). Circulating levels of eosinophil granule proteins have also been demonstrated to correlate with some aspects of disease activity. Therefore, in addition to simply measuring blood eosinophil counts, assessing eosinophil activation status may have clinical utility. Indeed, it has been reported that serum ECP and EPX levels predicted asthma risk more accurately than standard blood eosinophil counts in patients with allergic rhinitis (69).

**Figure 3:**
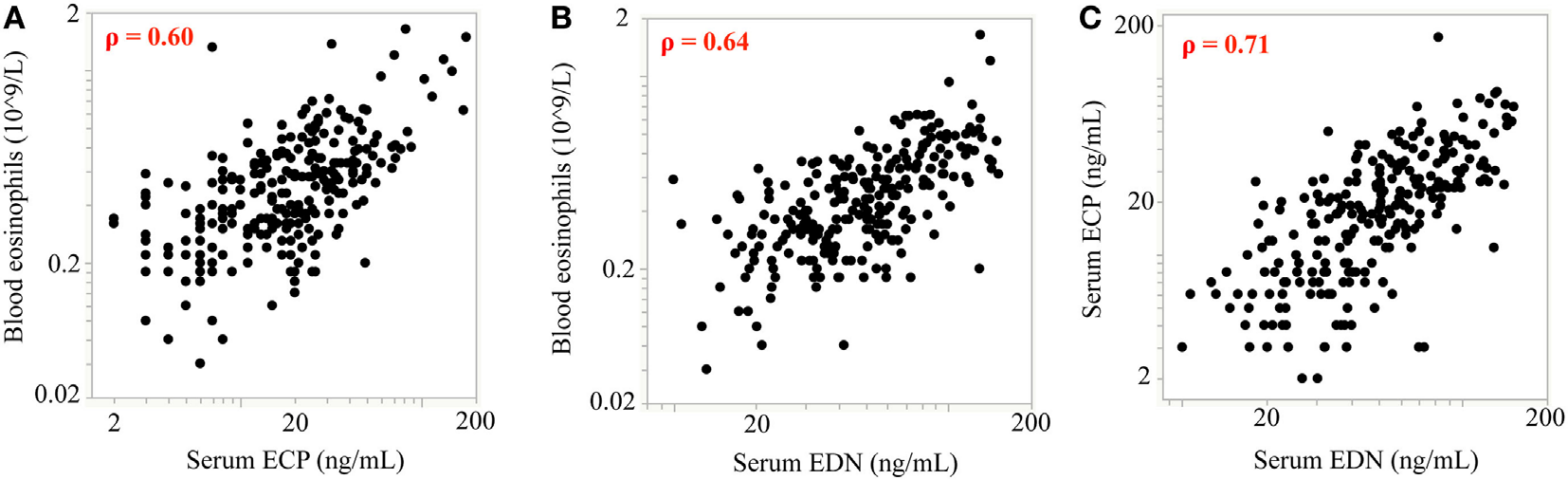
Baseline measurements of blood eosinophil counts are positively correlated with eosinophil cationic protein (ECP) and eosinophil-derived neurotoxin (EDN) levels. At baseline, there was a strong positive intercorrelation between blood eosinophils and **(A)** serum ECP and **(B)** serum EDN. **(C)** ECP and EDN levels were also strongly positively correlated. Spearman’s correlation coefficient was employed for statistical analysis. For all correlations *p* < 0.0001; rho (ρ) values are shown on each plot.

**Figure 4:**
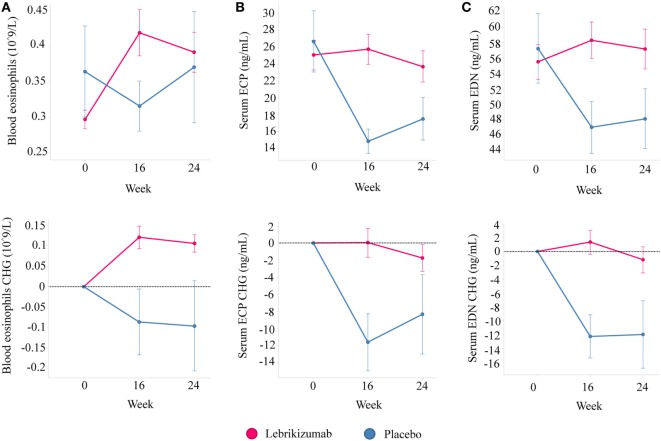
Lebrikizumab treatment increases peripheral blood eosinophil counts but circulating levels of eosinophil cationic protein (ECP) and eosinophil-derived neurotoxin (EDN) remain unchanged. **(A)** Absolute levels and changes from baseline show increased blood eosinophils in patients treated with lebrikizumab at 16 and 24 weeks [mean change of 0.12 × 10^9^/L (95% CI 0.08–0.16) and 0.10 × 10^9^/L (95% CI 0.06–0.15) respectively, *p* < 0.001] but no significant change in the placebo arm was observed. **(B)** Absolute levels and changes from baseline show no change in serum ECP in patients treated with lebrikizumab but a decrease in patients in the placebo arm at 16 and 24 weeks [mean change of −11.59 ng/mL (95% CI −18.80 to −4.38), *p* = 0.018 and −8.31 ng/mL (95% CI −16.71 to 0.08), *p* = 0.052 respectively]. **(C)** Absolute levels and changes from baseline show no change in serum EDN in patients treated with lebrikizumab but a decrease in patients in the placebo arm at 16 and 24 weeks [mean change of −12.25 ng/mL (95% CI −19.33 to −5.17), *p* = 0.0008 and −11.94 ng/mL (95% CI −20.25 to −3.64), *p* = 0.0051, respectively]. Graphs show mean ± SE and paired Student’s *t*-test were carried out (CHG, change).

Of note, treatments targeting the IL-13 pathway have consistently reported increases in blood eosinophil counts. However, the activation status of the eosinophils has not been characterized. To address this we analyzed serum levels of two eosinophil granule proteins, ECP and EDN, using pooled data from two independent Phase 2 studies investigating the efficacy of lebrikizumab (an anti-IL-13 monoclonal antibody) in patients with uncontrolled asthma despite maintenance therapy with inhaled corticosteroids (ICS) and a second controller. All patients provided written informed consent for their samples to be used for research purposes. Patients received either placebo or lebrikizumab (37.5, 125, or 250 mg) (47). Blood eosinophils, FeNO, and serum periostin were measured during this study, and pharmacodynamic (PD) effects were observed on each of these biomarkers. Blood eosinophil counts increased in response to treatment, and there was a slight trend toward a dose response. FeNO and serum periostin levels decreased after treatment but this was not dose dependent. Given the similar PD effects across treatment arms they were combined to analyze the eosinophil activation status after lebrikizumab treatment. Serum levels of ECP and EDN were measured at baseline and after 16 and 24 weeks of placebo or lebrikizumab treatment from a subset of patients who had comparable baseline characteristics to the overall study population (Table 1). These previously unpublished data demonstrated that at baseline there was a strong positive intercorrelation between blood eosinophils and serum ECP and EDN, suggesting that serum ECP and EDN may be secreted from blood eosinophils (Figure 3). Patients treated with lebrikizumab had increased blood eosinophil numbers after 16 and 24 weeks of treatment. However, serum ECP and EDN levels remained unchanged suggesting that while lebrikizumab treatment led to increased blood eosinophil levels it did not result in blood eosinophil activation (Figure 4). Of note, serum ECP and EDN levels in the placebo group significantly declined at 16 and 24 weeks. This decrease was unexpected given the unchanged blood eosinophil numbers over time in the placebo arm and therefore needs to be further explored.

**Table 1:** Summary of key patient characteristics at baseline.

	Placebo (*n* = 64)	Lebrikizumab (*n* = 191)
Age, mean (SD), years	48.9 (13.7)	48.4 (12.8)
Female, *n* (%)	41 (64.1)	112 (58.6)
Baseline ICS dose ≥1,000 μg/day, *n* (%)	14 (21.9)	65 (34.0)
Pre-bronchodilator FEV_1_ (% of predicted), mean (SD)	61.3 (10.7)	62.5 (10.2)
Time in placebo-controlled period, median (range), weeks	28.6 (19.0–48.1)	32.1 (19.3–49.0)
Blood eosinophils, mean (SD), 10^9/L	0.32 (0.29)	0.30 (0.27)
ECP, mean (SD), ng/mL	27 (29)	25 (24)
EDN, mean (SD), ng/mL	57.2 (35.4)	55.7 (30.6)

*There were no significant differences in these characteristics between placebo and lebrikizumab treatment arms*.

*ICS, inhaled corticosteroids; FEV_1_, forced expiratory volume in 1 second; ECP, eosinophil cationic protein; EDN, eosinophil-derived neurotoxin*.

While these data are potentially interesting and begin to shed light on the relationship between IL-13 and eosinophils in asthma there are some caveats. This was a *post hoc* analysis carried out in a subset of patients from two independent Phase 2 studies and therefore will require further validation to better understand the role of IL-13 in the number and activation status of eosinophils in asthma. Further investigation is also required to definitively address what are the consequences, if any, of increased blood eosinophils in asthma patients in response to anti-IL-13 therapies.

### Targeting IL-13 in Asthma

Moderate to severe asthma patients with poorly controlled disease represent a substantial unmet medical need. Compelling evidence for the role of IL-4 and IL-13 in driving type 2/eosinophilic asthma has led to the development of several therapeutic candidates to target these pathways (Figure 1; Table 2). Lebrikizumab is a humanized monoclonal antibody that binds to soluble IL-13 with high affinity and blocks signaling through the IL-4Rα/IL-13Rα1 heterodimer. Phase 2 clinical trials assessing lebrikizumab in moderate to severe uncontrolled asthma showed that treatment significantly improved lung function (49) and reduced the rate of exacerbations (47), compared to placebo. However, in two Phase 3 studies conducted in parallel there were inconsistent results; LAVOLTA I reported a significant reduction in exacerbations in lebrikizumab-treated patients compared to placebo but this did not replicate in LAVOLTA II. However, lung function as determined by forced expiratory volume in 1 second (FEV_1_) improvement was observed in both studies (70). Tralokinumab, a human IL-13-neutralizing monoclonal antibody blocking binding of IL-13 to both IL-13Rα1 and IL-13Rα2, has been assessed in clinical trials of moderate to severe uncontrolled asthma patients. A Phase 2 study investigating different dosing regimens of tralokinumab reported a trend for improved lung function after 16 weeks of treatment, but no change in asthma control questionnaire (ACQ)-6 score (71). A subsequent Phase 2b trial investigating two and four weekly dose regimens found that there was no significant reduction in asthma exacerbation rates but patients dosed every 2 weeks showed an improvement in lung function (48). Phase 3 studies to evaluate the efficacy and safety of tralokinumab in adults and adolescents with inadequately controlled asthma are currently underway (NCT02161757, NCT02449473, and NCT02281357). Dupilumab, a humanized monoclonal antibody to IL-4Rα that inhibits both IL-4 and IL-13 signaling, is being assessed in patients with uncontrolled asthma. An initial study evaluating the efficacy of dupilumab was carried out in persistent, moderate-to-severe asthma patients with elevated eosinophil levels (blood eosinophil count of at least 300 cells/mL or sputum eosinophil levels of at least 3%). In this study, patients on background ICS and long-acting beta-agonist (LABA) therapy were randomized to receive dupilumab or placebo and background treatment was withdrawn. In this context, dupilumab treatment led to a reduction of asthma exacerbations and improved lung function compared to placebo (50). A pivotal Phase 2b 24-week study in persistent, moderate-to-severe asthmatics on background ICS in which patients were enrolled irrespective of baseline eosinophil levels reported lung function improvement and a relative annualized exacerbation rate reduction in both eosinophil high and low patients. However, the lung function improvement and absolute exacerbation rate reduction were greater in the eosinophil high group (72). Phase 3 studies to evaluate the efficacy and safety of dupilumab in patients with persistent asthma (NCT02414854 and NCT02528214) are ongoing.

**Table 2:** IL-13-targeted therapies in eosinophilic diseases.

Drug (company)	Mechanism of blocking IL-13	Disease—target patients	Clinical trial outcome
Lebrikizumab (Roche)	Binds soluble IL-13 blocking IL-13Rα1 signaling	Asthma—moderate to severe AD—moderate to severe	Phase III—inconsistent results across 2 studies. Study 1—reduced exacerbations and improved FEV_1_. Study 2—no statistical significant effect on exacerbations but improved FEV_1_ (60) Ongoing Phase II trial
Tralokinumab (Medimmune/AstraZeneca)	Binds soluble IL-13 blocking both IL-13Rα1 and IL-13Rα2 signaling	Asthma—moderate to severe AD—moderate to severe	Phase IIb—no effect on exacerbations but improved FEV_1_ (48) Ongoing Phase III trials Phase IIb—improvement in EASI, SCORAD and DLQI
Dupilumab (Regeneron/Sanofi)	Binds IL-4Rα blocking both IL-4 and IL-13 signaling	Asthma—uncontrolled AD—moderate to severe EoE—active, moderate to severe CRSwNP—refractory to intranasal corticosteroids	Phase IIb—reduced exacerbations and improved FEV_1_ (62) Ongoing Phase III trial Phase III—improved EASI, IGA and symptoms of depression and anxiety (95) Ongoing phase II trial Phase II—reduced endoscopic nasal polyp burden (125) Ongoing Phase III trial
QAX576 (Novartis)	Monoclonal antibody binding to IL-13	EoE	Primary end point not met but decreased esophageal eosinophil counts
RPC4046 (Celgene)	Blocking both IL-13Rα1 and IL-13Rα2	EoE	Ongoing Phase II trial

*IL, interleukin; FEV_1_, forced expiratory volume in 1 second; AD, atopic dermatitis; EASI, Eczema Area Severity Index; SCORAD, Scoring Atopic Dermatitis; DLQI, Dermatology Life Quality Index; EoE, eosinophilic esophagitis; CRSwNP, chronic rhinosinusitis with nasal polyps; IGA, Investigator’s Global Assessment*.

While clinical trials of lebrikizumab, tralokinumab, and dupilumab targeting IL-13 had an acceptable overall safety profile, increases in blood eosinophil numbers were reported for each intervention (48, 70, 72). It is hypothesized that elevated blood eosinophil levels may be a result of reduced trafficking of eosinophils from the circulation to the airway and/or other tissues, where they can exert their pathogenic effects, due to decreased expression of IL-13-induced chemokines. Indeed, unpublished data from our preclinical studies testing the efficacy of anti-IL-13 in a mouse model of asthma found this to be the case. C57/B6 mice were challenged with the house dust mite extract, *Dermatophagoides farinae*, and treated prophylactically with either anti-IL-13 or an isotype control antibody. Administration of anti-IL-13 resulted in decreased BAL eosinophilia compared to control, however, there was concomitant upregulation of blood eosinophils in the anti-IL-13-treated mice but not in the controls. Eosinophil dynamics upon treatment with anti-IL-13 and anti-IL-5 have also been studied using a mathematical model. The model incorporated levels of eotaxin and periostin as chemoattractants for eosinophils to the lung. It predicted that treatment with anti-IL-13 would result in a decrease in lung eosinophils and an increase of blood eosinophils while anti-IL-5 treatment would result in a decrease in both blood and airway eosinophils (73). Of note, treatment with the anti-IL-5 therapy, mepolizumab, consistently leads to decreased blood eosinophil levels. However, differential reductions in airway eosinophils have been observed depending on which compartment of the lung is being sampled. Sputum eosinophil levels decreased significantly in response to mepolizumab treatment but tissue eosinophil numbers did not (74, 75). Further investigation of eosinophil dynamics in humans is required to confirm these animal data and modeled predictions, and several studies are ongoing. To evaluate the effect of blocking IL-13 on human airway eosinophil dynamics, studies with lebrikizumab (NCT02099656) and tralokinumab (NCT02449473) are being conducted in inadequately controlled asthmatics. In addition, the effect on inflammatory cells in the airway after blocking IL-4 and IL-13 signaling by dupilumab is being examined in patients with persistent asthma (NCT02573233). The results of these studies should shed significant light on the relationships between IL-13 and airway eosinophilia and other pathologies in asthma patients *in vivo*.

## Atopic Dermatitis

Atopic dermatitis is the most common recurring inflammatory skin disease in children, with an average world prevalence of 7.9% in 6–7-year olds (76). Disease manifestations include dry skin, eczematous lesions, intense pruritus, and high serum IgE levels. In AD, compromised epidermal barrier function leads to enhanced allergen penetration and systemic IgE sensitization. Patients with AD exhibit blood eosinophilia (77), eosinophil infiltrates in skin lesions (78), and deposition of eosinophilic granule proteins (79).

### IL-13 and Eosinophils in AD

Signatures of responsiveness to Th2, Th22, Th17, and Th1 cytokines are associated with AD skin at various stages of the disease. In particular, the Th2 cytokines IL-4 and IL-13 have been shown to play central roles in AD by modulating the epidermal barrier, including suppression of keratinocyte epidermal differentiation complex (EDC) genes (80) and inhibition of antimicrobial peptide production (81, 82). IL-13 mRNA has been shown to positively correlate with AD disease severity in acute and chronic lesional skin (83–85). Patients with AD have higher levels of serum IL-13 compared to healthy controls (58, 86). Children with more severe AD exhibited a higher percentage of IL-13-expressing CD4^+^ T cells in peripheral blood (87). *In vitro* treatment of normal human epidermal keratinocytes with IL-13 led to increased expression of a key chemokine for eosinophil recruitment, CCL26 (88). In mice, transgenic overexpression of IL-13 in the skin induced key features of AD, including pruritus, elevated IgE, and eosinophilic infiltration. There were also elevated levels of eosinophil chemoattractants such as CCL11 in the skin, driving recruitment of eosinophils from the blood to the tissue. This established a clear role for IL-13 in AD (89). IL-4 and IL-13 share overlapping biological functions and pathophysiological roles in AD in part due to the shared use of the IL-4Rα/IL-13Rα1 receptor complex and subsequent signaling through STAT6. Mice constitutively expressing active STAT6 (Stat6VT) were found to develop spontaneous AD-like disease with decreased EDC gene expression and increased IL-4, IL-13, and eosinophils in the lesional skin. IL-4 deficiency in these mice (IL-4^−^/^−^ Stat6VT) attenuated development of allergic skin disease and eosinophilic inflammation, while therapeutic blockade with anti-IL-13 in the Stat6VT mice led to the rescue of EDC gene expression (90). Similarly, blockade of IL-13 by topical delivery of IL-13 antisense oligonucleotides reduced AD-related cytokines, IgE, and inflammatory cells in the skin in an epicutaneous OVA sensitization model (91). As described earlier, IL-13 also binds to IL-13Rα2, a decoy receptor that lacks an intracellular signaling motif and which may serve as a negative feedback regulator of IL-13 signaling. Keratinocytes from lesional skin of AD patients showed elevated expression of IL-13Rα2 (92). IL-13 also induced the expression of IL-13Rα2 in human keratinocytes in a STAT6-dependent manner (93). Consistent with its role as a decoy receptor, mice deficient for IL-13Rα2 showed increased transepidermal water loss, skin inflammation, peripheral eosinophilia, and IgE in a model of AD compared to control mice (94).

### Targeting IL-13 in AD

Given the strong biologic rationale various companies have moved forward with therapeutic candidates targeting type 2 cytokines in AD (Table 2). Dupilumab was investigated in patients with moderate to severe AD inadequately controlled by topical treatment. In two Phase 3 trials, dupilumab improved the signs and symptoms of AD, anxiety and depression, and quality of life compared to placebo (95). These results further validate the hypothesis that the type 2 cytokines IL-4 and IL-13 are key drivers of AD. Of note and similar to therapies targeting the IL-13 pathway in asthma, these trials reported elevated blood eosinophil levels in patients treated with dupilumab compared to placebo. Biologics specifically targeting IL-13 have completed Phase 2 studies for the treatment of AD. In an ongoing study, lebrikizumab was evaluated in patients with persistent, moderate to severe AD inadequately controlled by topical corticosteroids (TCS) (NCT02340234). Efficacy of tralokinumab was assessed in patients with moderate to severe AD on a background of TCS (NCT02347176) and showed statistically significant improvements in symptoms of AD. These results support a key role of IL-13 signaling in AD pathophysiology. However, due to the differences and limitations in trial designs, the relative contributions of IL-4 vs. IL-13 and a role of IL-13Rα2 in human AD could not be fully elucidated and will require further investigation.

## Eosinophilic Esophagitis

Eosinophilic esophagitis is a chronic inflammatory disease of the esophagus. It is one of the most common conditions diagnosed during the assessment of feeding problems in children and dysphagia and food impaction in adults (96). EoE occurs worldwide with increasing prevalence, currently at 0.4% in Western countries (97). The diagnosis of the EoE has been challenging. Two key components are required: (i) clinical symptoms including feeding problems, vomiting, and abdominal pain in children, and dysphagia and food impaction in adolescents and adults and (ii) histological evaluation of 15 or more eosinophils per high-powered field in esophageal mucosal biopsy following treatment with proton pump inhibitors (98).

### IL-13 and Eosinophils in EoE

Defective barrier function, evidenced by thickening of the mucosal basal layer, dilated interepithelial spaces and altered epithelial barrier function have been observed in esophageal tissues from patients with EoE (99). The resulting increased epithelial permeability is believed in turn to enhance antigen presentation and eosinophil recruitment. Both environmental and genetic predispositions modulate immune responses that play an important role in the pathogenesis of EoE. Type 2 responses induced primarily by food antigens have been thought to be a major driver of the disease. In particular, IL-13 is overexpressed in biopsies from patients with EoE (100). IL-13 has been shown to affect epithelial barrier function by downregulating EDC genes such as filaggrin (*FLG)* (101). *In vitro* studies found that IL-13 upregulates IL-5, CCL26, and other related cytokines that contribute to eosinophilia (101, 102). Furthermore, a genome-wide association study (GWAS) revealed a potent IL-13 inducer, *TSLP*, and IL-13 downstream response genes, *CCL26* and calpain 14 (*CAPN14*) were associated with EoE (102–105). EoE transcriptome signatures identified by microarray and RNA-seq analyses for dysregulated genes in the esophagi of patients with EoE revealed a significant involvement of IL-13 and exhibited a striking degree of overlap with the gene expression pattern observed in endobronchial biopsies of “Th2-high” asthma patients (106–108). The long non-coding RNA *BANCR* is induced by IL-13 and its expression correlates with levels of eosinophils and transcripts known to be involved in EoE pathogenesis. Another transcriptional target of IL-13, neurotropic tyrosine kinase receptor type 1 (NTRK1), was upregulated in EoE esophageal tissues. This upregulation is believed to cause enhanced responsiveness of epithelial cells to NGF, a ligand of NTRK1, and the subsequent induction of the eosinophil chemokine, CCL26 (109).

### Targeting IL-13 in EoE

Several biologics targeting IL-13 have been tested in clinical trials for the treatment of EoE (Table 2). QAX576, an anti-IL-13 monoclonal antibody, was tested in a small cohort of patients with proton pump inhibitor-resistant EoE for its efficacy in reducing peak eosinophil counts in the esophageal tissue after 8 weeks of treatment. The primary end point was not met; nevertheless, QAX576 reduced esophageal eosinophil counts by 60% compared to an increase of 23% with placebo and there was a slight trend toward improved symptoms. Transcriptomics were also carried out on biopsy specimens collected on day 85 of the study and showed that EoE-related genes were downregulated, including the eosinophil chemoattractant CCL26, which suggests that IL-13 is a significant driver of the differential gene expression observed in EoE. QAX576 had no effect on blood eosinophil counts (110). RPC4046, an anti-IL-13 monoclonal antibody that blocks both IL-13Rα1 and IL-13Rα2, is currently being studied in a dose ranging Phase 2 study in EoE with mean eosinophil count as a primary outcome (NCT02098473). The efficacy of dupilumab is also being investigated in a Phase 2 trial in patients with active, moderate to severe EoE. However, the primary outcome for this trial is change in the Straumann Dysphagia Instrument (SDI) patient-reported outcome (PRO) score and changes in eosinophil counts will be evaluated as one of the secondary end points (NCT02379052).

## CRS with Nasal Polyps

Chronic rhinosinusitis is an inflammatory pathological condition of the nose and paranasal sinuses. Patients with CRS are characterized by nasal obstruction, drainage, compromised olfaction, and prolonged facial pain or pressure (111). CRS is classified into two subtypes: CRS without nasal polyps (CRSsNP) and CRS with nasal polyps (CRSwNP) (112). In the United States and Europe, the majority of CRSwNP patients have significant eosinophilic infiltration in their polyp tissue (113).

### IL-13 and Eosinophils in CRSwNP

Eosinophilic CRSwNP (ECRSwNP) has been increasing in prevalence worldwide, estimated to be 2.1%–2.7% in adults (114–116). It represents a recalcitrant form of the disease resistant to medical or surgical intervention (113). ECRSwNP is characterized by type 2 inflammation with elevated levels of IL-5, IL-13, and eosinophils in the polyp tissue (117). Th2 cells, ILC2s, mast cells, and eosinophils are hypothesized to be the major sources of type 2 cytokines in ECRSwNP. Similar to asthma, AD, and EoE, it is believed that IL-4 and IL-13 play important roles in the pathophysiology of ECRSwNP. IL-13 has been shown to affect the integrity of the sinonasal epithelial barrier by inhibiting the expression of tight junction proteins (118) and antimicrobial peptide production (119). The expression of eosinophil chemoattractants, such as CCL11, CCL24, and CCL26, are elevated in ECRSwNP (120–122). *In vitro*, IL-4 or IL-13 in combination with TNF induced elevated expression of CCL11 in fibroblasts and airway epithelial cells derived from these patients, suggesting a positive feedback loop between eosinophil recruitment and type 2 inflammation (123). Transcriptomic analysis of RNA-seq data comparing nasal polyps from eosinophilic and non-ECRSwNP and nasal mucosa from control subjects revealed distinct expression profiles between these subgroups. Notably, IL-13 and CCL26 are specifically overexpressed in ECRSwNP, along with other chemokines that mediate eosinophilic inflammation (124).

### Targeting IL-13 in Nasal Polyps

There are currently no biologic therapies approved for the treatment of nasal polyps. Dupilumab has been evaluated in a Phase 2 clinical trial for its efficacy in patients with chronic sinusitis and nasal polyposis refractory to intranasal corticosteroids and showed encouraging results (125). Patients who received dupilumab together with mometasone furoate nasal spray experienced reduced endoscopic nasal polyp burden compared to patients that received mometasone plus placebo (Table 2). Longitudinal analysis of plasma CCL26 showed a decrease after dupilumab treatment, while there was a trend toward decreased serum CCL17 levels. A transient increase in blood eosinophils was observed in response to treatment in some patients. Phase 3 clinical studies are currently underway (NCT02912468, NCT02898454).

## Conclusion

Asthma and other eosinophilic disorders such as AD, EoE, and CRSwNP are highly prevalent and numbers of people affected are continuing to increase. There are subsets of patients with each of these conditions that do not respond adequately to standard of care, experience significant morbidity, consume substantial healthcare expenditures, and thus represent an unmet clinical need.

Molecular phenotyping has been instrumental in understanding the underlying biology of these diseases. As such, we are now beginning to understand the roles of various cytokines and immune effector cells, in particular type 2 cytokines and eosinophils. IL-5 was identified as the key driver of eosinophilopoiesis, leading to the development of multiple therapies targeting IL-5 and eosinophils. The efficacy of these drugs indicates the vital role of eosinophils in diseases such as asthma. In addition, substantial evidence exists from *in vitro* cell culture models, *in vivo* animal models and observational human studies for the role of IL-13 (and IL-4) in driving eosinophilia and type 2 inflammation, which led to interventional studies targeting these pathways in human disease. Biologics targeting the IL-13 pathway have demonstrated efficacy, particularly in subsets of patients with evidence of eosinophilic disease. Ongoing clinical trials will help to further dissect the contributions of IL-13 to tissue eosinophilia. Notably, blood eosinophil counts themselves are used as a biomarker in many of these clinical studies. Eosinophil “high” patients have experienced greater clinical benefit from anti-type 2 therapies compared to eosinophil “low” patients. This further necessitates the need to fully understand the role of eosinophils in type 2 driven diseases, as well as define the mechanisms contributing to disease pathology in patients with low levels of type 2 inflammation and eosinophils.

The advent of better mouse models and ongoing clinical trials targeting multiple pathways, including IL-13, will help garner a better understanding of eosinophil biology and improve therapeutic strategies for treating eosinophilic disorders in the future.

## Author Contributions

ED, CH, KW, and JA made substantial contributions to important intellectual content, drafting of the manuscript and revisions. FC and JB also carried out experiments, analyzed and interpreted data. All authors approved the final version of the manuscript.

## Conflict of Interest Statement

The authors are employees of Genentech, Inc., a member of the Roche Group, and may have an equity interest in Roche or are named as coinventors on patent applications related to the development of biologic therapies and biomarkers for the treatment of asthma. The authors have no other relevant affiliations or financial involvement with any organization or entity with a financial interest in or financial conflict with the subject matter or materials discussed in the manuscript apart from those disclosed.
